# Pre-processing and differential expression analysis of Agilent microRNA arrays using the *AgiMicroRna *Bioconductor library

**DOI:** 10.1186/1471-2164-12-64

**Published:** 2011-01-26

**Authors:** Pedro López-Romero

**Affiliations:** 1 Epidemiology, Atherothrombosis and Imaging Department. Centro Nacional de Investigaciones Cardiovasculares Carlos III (CNIC), Melchor Fernández Almagro 3, E-28029 Madrid, Spain

## Abstract

**Background:**

The main research tool for identifying microRNAs involved in specific cellular processes is gene expression profiling using microarray technology. Agilent is one of the major producers of microRNA arrays, and microarray data are commonly analyzed by using R and the functions and packages collected in the Bioconductor project. However, an analytical package that integrates the specific characteristics of microRNA Agilent arrays has been lacking.

**Results:**

This report presents the new bioinformatic tool *AgiMicroRNA *for the pre-processing and differential expression analysis of Agilent microRNA array data. The software is implemented in the open-source statistical scripting language R and is integrated in the Bioconductor project (http://www.bioconductor.org) under the GPL license. For the pre-processing of the microRNA signal, *AgiMicroRNA *incorporates the *robust multiarray average algorithm*, a method that produces a summary measure of the microRNA expression using a linear model that takes into account the probe affinity effect. To obtain a normalized microRNA signal useful for the statistical analysis, *AgiMicroRna *offers the possibility of employing either the processed signal estimated by the *robust multiarray average algorithm *or the processed signal produced by the Agilent image analysis software. The *AgiMicroRNA *package also incorporates different graphical utilities to assess the quality of the data. *AgiMicroRna *uses the linear model features implemented in the *limma *package to assess the differential expression between different experimental conditions and provides links to the *miRBase *for those microRNAs that have been declared as significant in the statistical analysis.

**Conclusions:**

*AgiMicroRna *is a rational collection of Bioconductor functions that have been wrapped into specific functions in order to ease and systematize the pre-processing and statistical analysis of Agilent microRNA data. The development of this package contributes to the Bioconductor project filling the gap in microRNA array data analysis.

## Background

MicroRNAs are a family of small single-stranded non-coding RNAs which regulate gene expression [1]. Functional studies show that microRNAs participate in virtually every cellular process investigated, and changes in their expression might underlie many human diseases [2]. One of the commercial microarrays for microRNA profiling has been developed by *Agilent Technologies. *Agilent microarrays use a single-color array protocol and combine a direct labelling method with innovatively designed, in situ-synthesized probes that have minimal sequence bias [3]. The efficient labelling method means that the Agilent platform only requires 100 ng of input total RNA and does not require size fractionation or amplification steps that could introduce bias during microRNA profiling. This results in optimal sensitivity and specificity for both sequence and size discrimination, even between highly homologous mature microRNAs.

For each microRNA, the Agilent microRNA microarray platform makes measurements with a number of different oligonucleotide probes that are replicated a number of times across the array surface. These replicate signals are summarized into a total gene signal (TGS) with the proprietary *Agilent Feature Extraction *(AFE) image analysis algorithm [4], which makes use of the background corrected signals. This TGS could be used for the differential expression analysis without further pre-processing adjustments; however, different studies indicate that normalization of the microRNA data between arrays improves both sensitivity and specificity in comparison with non-normalized data. For example, Rao et al. [5] studied the performance of different normalization methods, using custom single-color microRNA data, and concluded that the quantile method [6,7] works much better than no normalization in terms of bias. Pradervand et al. [8] carried out a similar investigation using the Agilent microRNA microarray platform and the summarized TGS obtained with the AFE image analysis software provided by the vendor. These authors proposed a novel normalization method based on the selection of invariant microRNAs that were subsequently used to normalize the arrays. This invariant normalization method was compared with other normalization routines such as scale, quantile [6,7] and VSN [9], and the authors concluded that all normalization methods performed better than no normalization, with invariant and quantile normalization being the most robust [8]. In addition to the availability of different methods for data normalization between arrays, another pre-processing step that can be tackled in different ways is the summarization of probe level data into a single microRNA signal. For example, one alternative to the TGS computed by AFE is the *robust multiarray average *(RMA) proposed by Irizarry et al. [10] for the pre-processing of *Affymetrix *microarray data [11]. The RMA algorithm estimates a total gene signal by fitting a linear model to the probe data that takes into account the probe affinity effect. When applied to *Affymetrix *microarray data, the RMA protocol improved the accuracy and precision of expression measurements compared with other methods that summarized the multiple probe level data into a gene expression measure [10].

We previously applied the RMA algorithm to Agilent microRNA array data and compared the total gene signal estimated by RMA and by AFE algorithms, with both RMA-TGS and AFE-TGS signals normalized between arrays by quantile [12]. In that work we concluded that the RMA of the non background-corrected signal and the AFE-TGS normalized by quantiles were almost equally precise, although the RMA seemed to produce signals of lower variability at low intensity values, so the use of the RMA algorithm might be advantageous for the detection of low expressed genes, if it used with a non-background corrected signal. According to [12], either TGS using quantiles or RMA with no background correction are reasonable alternatives for the pre-processing of the microRNA Agilent data. We have included these pre-processing algorithms in *AgiMicroRna*, a new library of functions that have been conceived for the pre-processing and differential expression analysis of Agilent microRNA array data. A complete manual explaining all the technical details and guidelines to test the package functionality is provided with the software, so here we give a general description of its capabilities.

## Implementation

*AgiMicroRna *includes functions that wrap around already existing Bioconductor functions as well as new specific functions that have been collected and integrated into a library to facilitate the pre-processing and differential expression analysis of Agilent microRNA microarray data in a systematic and easy way. The package is written in R [13], open-source software for statistical computing, and is integrated in the Bioconductor project [14] under the GPL license.

To use the *AgiMicroRna *package, first download and start R 2.12.0 (or a more recent version) and install the Bioconductor basic libraries. Then we need to install the *AgiMicroRna 2.0.1 *or a more recent version of the library. This installation can be done by opening an R command window and entering the following commands: *source("http://bioconductor.org/biocLite.R") *and *biocLite("AgiMicroRna")*. Finally, to load the package into the current R session, type *library("AgiMicroRna")*. It might be advisable at this point to use the R command *sessionInfo() *to check that actually we have loaded the *AgiMicroRna 2.0.1 *library version (or a more recent version). *AgiMicroRna *includes a complete example data set together with a manual that provides full technical details of the functions included in the package and helps users to become familiar with its utilities. The functionality of the package can also be tested with other data sets deposited in the Gene Expression Omnibus (GEO) data base [15] with accession numbers GSE16444 and GSE15144. Both of these data sets are based on the Agilent Human microRNA Microarray 2.0 G4470B platform. As Additional file 1, we have included two R scripts to assist potential users with the use of the package.

### Input files

#### Target file

*AgiMicroRna *has been designed primarily to process data for analysis with the *limma *package [16], and a target file is needed in order to assign each scanned data file to its corresponding experimental group. The target file should be a tab delimited text format file, created by the user, in which the factors to be used in the statistical analysis are specified. The target file must contain the following columns: a first column *FileName*, including the image data file names; a second column *Treatment*, including the treatment effect or experimental group; and a third column *GErep *that assigns an integer to each treatment effect. Users can include additional columns containing information about other explanatory variables related to the specific experimental design. Once the library has been loaded in the R session, the target file associated with the example data included in the library can be accessed by typing *data(targets.micro) *in a command window. The target file for this example is shown in Table 1.

**Table 1:** Target file associated with the data example included in *AgiMicroRna*.

FileName	Treatment	GErep	Subject
mscA1.txt	A	1	1

mscA2.txt	A	1	2

mscB1.txt	B	2	1

mscB2.txt	B	2	2

mscC1.txt	C	3	1

mscC2.txt	C	3	2

#### Data files

*AgiMicroRna *typically reads the scanned data exported by the AFE image analysis software into R, and stores all the relevant information needed for the pre-processing steps in a specific R object of a class *uRNAList *specifically designed by the AgiMicroRna library. This *uRNAList *is a new defined R class similar to the class *RGList *that is used by the *limma *library [16], which uses names that are more appropriate to the Agilent microRNA data. In order to illustrate the functionality of the package, *AgiMicroRna *includes a typical *uRNAList *object that contains a data example with all the information that is needed for the data pre-processing and subsequent differential expression analysis using the *AgiMicroRna *package. This particular data example includes information from 6 human microRNA Agilent arrays (Agilent Human microRNA Microarray 2.0 G4470B, Agilent Technologies) and reproduces exactly what *AgiMicroRna *would read into R from the AFE output data files. These data have been obtained from the GEO data base [15] (accession number GSE19232) (see Results section for a more detailed description). Typing *data(dd.micro) *in the R command window loads the *uRNAList *containing the data example into the current R session, allowing inspection of the information that is loaded from the AFE output files into a *uRNAList *object. The components stored in this *uRNAList *object are shown in Table 2, and a detailed description is given in the help files included in the package.

**Table 2:** Variables stored in the *uRNAList *object retrieved from the AFE scanned data files.

Variable Name	Description
***gMeanSignal***	Raw signal.

***gProcessedSignal***	Signal obtained after all the AFE processing steps (background correction) have been completed. Typically it contains the *Multiplicatively Detrended BackgroundSubtracted Signal *or the *BackgroundSubtractedSignal*.

***gTotalProbeSignal***	Average of all the background corrected signals (gProcessedSignal) for each replicated probe (features with the same sequence) multiplied by the total number of probe replicates.

***gTotalGeneSignal***	Sum of the *TotalProbeSignal *over the number of probes per gene.

***targets***	Contains the name of the AFE scanned data files as specified in the target file.

***ProbeName***	Agilent-assigned identifier for the probe synthesized on the microarray.

***GeneName***	Systematic name for the gene for which the probe provides expression information.

***ControlType***	Specifies whether the feature is a microRNA gene or a control (0 = gene feature, +1 = positive control, -1 = negative control)

***gIsGeneDetected***	Boolean variable that informs if the gene was detected on the microarray. A feature is considered detected if the signal is three fold greater than the error. If one probe of the probe set for a gene is detected, the gene is considered detected (1 = is detected 0 = is not detected).

***gIsSaturated***	Boolean flag. 1 indicates that the feature is saturated, i.e. at least half of the inlier pixels in the feature have intensities above the saturation threshold defined by AFE.

***gIsFeatNonUnifOL***	Boolean flag. 1 indicates that the feature is a non-uniformity outlier; the measured feature pixel variance is greater than the expected feature pixel variance plus the confidence interval.

***gIsFeatPopnOL***	Boolean flag. 1 indicates that the feature is a population outlier.

***gBGMedianSignal***	Median raw signal of the local background calculated from intensities of all inlier pixels that represent the local background of the feature.

***gBGUsed***	Background signal used by AFE algorithms to obtain the background corrected signal. Usually the *gBGUsed *is the sum of the local background plus the spatial detrending surface value. The spatial detrend surface value estimates the noise due to a systematic gradient on the array.

### Data quality assessment tools

#### Plotting Functions

The quality of the data can be evaluated using standard graphics utilities included in *AgiMicroRna*, such as boxplots, density plots, MA plots and relative log expression plots [17]. The MA plots represent the fold-change (M) in the y-axis against the average log expression (A) for two given arrays. To reduce the number of pairwise comparisons, *AgiMicroRNA *compares all arrays with a reference array. The signal for each spot on the reference array is computed as the median of the corresponding spots in all arrays. The relative log expression plot displays, for each sample, a boxplot of the relative log expression (RLE). The RLE for every spot in the array is computed as the difference between that spot and the same spot in the reference array. Since the majority of the spots are expected not to be differentially expressed, the RLE boxplots should be centred on zero and show approximately the same dispersion. *AgiMicroRna *also hierarchically clusters the samples using the *hclust *function of the R *stats *package. This hierarchical clustering can use either the whole set of genes or a reduced set defined by the user. Some caution must be taken in the interpretation of these clusters. The variables that distinguish the experimental conditions from one another are mainly the differentially expressed genes, and the number of these genes relative to the full set of genes in the data set is normally low. Therefore cluster analysis will often not reflect the influence of these relevant genes in the grouping of the samples, and the cluster plot will mainly show other grouping aspects, or simply random noise.

#### Array reproducibility

In the Agilent microRNA platforms each microRNA gene is normally interrogated by 16 probes, using either 2 probes replicated 8 times or 4 probes replicated 4 times. *AgiMicroRna *uses this probe replication to compute the coefficient of variation (CV) for each array. A lower CV-array indicates better reproducibility.

### Data pre-processing

**Figure 1:**
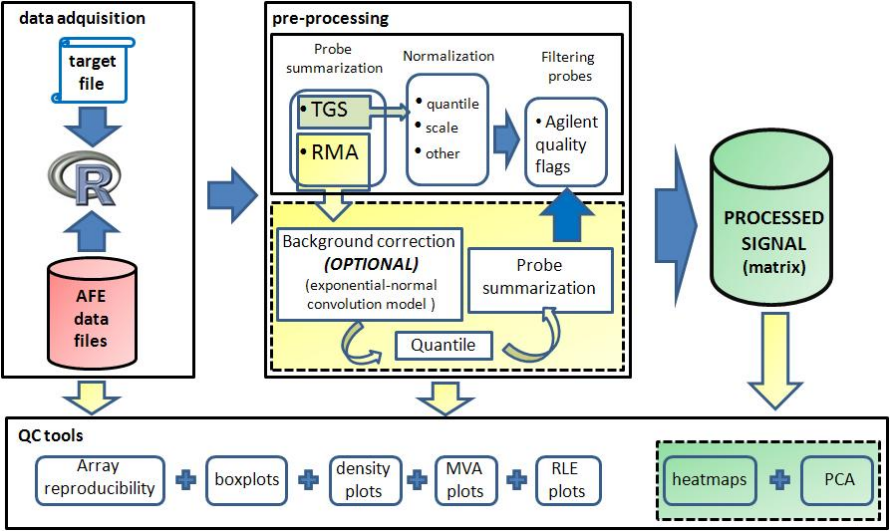
**Pre-processing steps**. *AgiMicroRna *includes two distinct pre-processing protocols for transforming the raw probe level data into the processed data that contain the summarized and normalized microRNA gene signals. The first protocol comprises the following steps: 1) acquisition of the TGS processed by AFE, and 2) normalization between arrays by scale or quantile methods. The second option uses the RMA algorithm via the following steps: 1) the raw mean signal can be background corrected (optional, see recommendations in the text); 2) the signal is normalized between arrays by quantile normalization, and 3) the probe level data is summarized into a single microRNA measure. Selected microRNAs can be filtered out according to the Flags assigned to each probe by the Agilent Extraction Software.

Agilent microRNA microarrays interrogate each microRNA gene with different probe sets. To make statistical inferences, a summary expression measure for each microRNA, possibly normalized between arrays, is needed. *AgiMicroRna *includes two alternative strategies for pre-processing the raw probe level data to yield a summarized and normalized microRNA gene signal (Figure 1). The first approach is based simply on normalization of the AFE-processed TGS between arrays. The AFE-processed TGS is a background-subtracted signal and hence might contain negative values. Therefore to obtain positive values before log transformation, *AgiMicroRna *either adds a small positive constant to all TGS signals or sets all negative TGS values to 0.5. This TGS signal can be used to make statistical inferences after a normalization step, either using the *quantile *or *scale *methods integrated in *AgiMicroRna *or any normalization method implemented in another Bioconductor package. The other approach incorporated in *AgiMicroRna *yields a summary microRNA gene measure using the RMA algorithm [10,12]. In the RMA algorithm implemented in *AgiMicroRna*, the signal can be first background corrected by fitting a normal + exponential convolution model to the vector of observed intensities [18]. When using the RMA algorithm, it might be a better option to omit background correction [12]. Whether or not the signal has been background corrected, the arrays are then normalized by quantile, and finally an estimate of the microRNA gene signal is obtained by fitting a linear model to the log2 transformed probe intensities. This model produces an estimate of the microRNA gene signal corrected for the probe effect.

#### Filtering Probes

The AFE image analysis software attaches a flag to each gene feature that identifies different signal quantification issues that can eventually be used to filter out microRNAs. *AgiMicroRna *includes filter functions to remove control features and to remove genes not detected in any of the experimental conditions under study. The filtering is done after normalization, and different arbitrary criteria can be established to be more or less demanding on the filtering of the genes. If the number of replicates is low it might be necessary to set more restrictive filtering limits to ensure that the comparisons between experimental conditions are done with a minimal amount of reliable information. For instance, each feature *i *in the sample *j*, *x*_*ij*_, has a corresponding FLAG *f*_*ij*_, that equals 1 *x*_*ij *_if is detected by AFE and 0 otherwise. For the filtering step, we set a limit L, which is the minimum percentage detection of a feature required in at least one experimental group for each feature. The filtering algorithm runs through all the features in the data set, and retains for further analysis features whose percentage of detection, under at least one experimental condition, is greater or equal than L. If for example, we have 4 replicates per experimental group, and we want to have at least 3 replicates for every feature detected in any of the experimental groups then we would set L = 75.

#### Differential expression analysis tools

**Figure 2:**
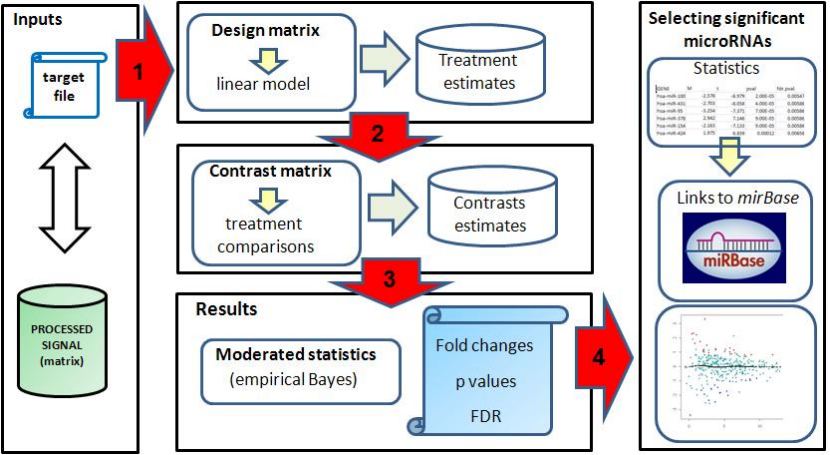
**Differential expression**. *AgiMicroRna *wraps the linear model features implemented in the *limma *package. The target file is used to define the factors to be used in the linear model that we are going to fit to each gene using the processed data. To specify the linear model we need to define a design matrix (step 1). A contrast matrix (step 2) is also needed to obtain the estimates of the contrast of interest. Moderated statistics are then obtained and the results are stored in a specific R object (step 3). Finally, specific functions implemented in *AgiMicroRna *are used to extract the results into separate output files (step 4).

The *AgiMicroRna *package makes statistical inferences about differential expression by using the linear model features implemented in the *limma *package [16]. The flow chart in Figure 2 indicates how differentially expressed genes are identified once the processed microRNA data have been generated. *AgiMicroRna *integrates different functions to extract useful information from the objects generated by *limma*, such as the list of microRNAs and associated statistics obtained from the differential expression analysis. The information given for each gene is shown in Table 3. The genes declared as significant are also listed in an html file that contains links to *miRBase *(http://microrna.sanger.ac.uk/) [19]. MA plots highlighting the differentially expressed genes are also generated.

**Table 3:** Statistics extracted after the differential expression analysis for every microRNA gene.

Statistic	Description
***Probe***	Probe name (one of the probes interrogating the gene)

***Gene***	microRNA gene name.

***M***	Fold change in log2 scale.

***A***	Mean of the intensity for that microRNA

***t***	Moderated t statistic of the contrast obtained by the *limma *function *eBayes()*

***pval***	p value of the t value. Degrees of freedom of the t distribution under the null hypothesis computed by *eBayes()*

***adj.pval***	p value adjusted by the selected *MTestmethod *option. If "none" were selected, this column will be the same as pval. If we have selected "BH", then, this column will be the same as in *fdr.pval.*

***fdr.pval***	p value adjusted by BH method

## Results

### RNA samples and experimental design

To demonstrate and test all the library functionalities we have created a data example using human microRNA expression data acquired from the GEO base [15] (accession number GSE19232). To obtain this expression data, and according to the information given by GEO, 100 ng of Cy3 labelled RNA were hybridized to Human miRNA V2 Microarray 8 × 15K (G4470B, Agilent Technologies) according to manufacturer's instructions (miRNA Microarray System Protocol, Agilent Technologies). Arrays were scanned at 5 um resolution on an Agilent DNA Microarray Scanner (G2565BA, Agilent Technologies) using the default settings for miRNA Microarray v2.0 (miRNA Microarray System Protocol, Agilent Technologies). Images provided by the scanner were analyzed using Agilent's software Feature Extraction version 9.5.3.1. [4], with default settings. The data example used in the library assumes three experimental conditions replicated two times and the goal is to compare two experimental treatments, MSC_B and MSC_C, with a control treatment MSC_A, assuming that each treatment was applied to cells obtained from the same subject (paired design). This example is used for illustrative purposes and is not intended to make any substantive biological sense.

### Agilent microRNA microarray

Agilent microRNA assays integrate eight individual microarrays on a single glass slide. Each microarray includes approximately 15 k features containing probes sourced from the *miRBASE *public database [19]. The probes are 60-mer oligonucleotides directly synthesized on the array. Agilent Human microRNA Microarray v2.0 contains 723 human and 76 human viral microRNAs, each of them replicated 16 times. There are 362 microRNAs interrogated by 2 different oligonucleotides, 45 microRNAs interrogated by 3, and 390 microRNAs interrogated by 4. Only 2 microRNAs are interrogated by a single oligonucleotide. The array also contains a set of positive and negative controls that are replicated a variety of times. Some of the positive controls are probes for non-microRNA human RNAs. These are replicated 20 times using 4 probes and the signals can be bright or dim depending on the sample. According to Agilent they do not appear to behave consistently enough to be used for normalization purposes.

### Array coefficient of variation

*AgiMicroRna *identifies the replicated probes (excluding control probes) for each feature in the array and computes the coefficient of variation (CV) for every probe set. The median CV for each probe set is reported as the array reproducibility.

### Agilent total gene signal

The AFE algorithms estimate a single intensity measure for each microRNA, referred to as total gene signal (TGS). This AFE-TGS is estimated as the *total probe signal *multiplied by the number of probes per gene. The *total probe signal *is the average of all the background subtracted signals for each replicated probe multiplied by the total number of probe replicates. Usually the background signal is the sum of the median local background signal plus the spatial detrending surface value computed by AFE, which estimates the noise due to a systematic gradient on the array. After this background correction, some microRNAs might show negative TGS values.

### Signal pre-processing

*AgiMicroRna *can use two protocols to generate the processed microRNA signal. The first uses the TGS computed by the AFE image analysis software, which is stored in the *RGList *as *gTotalGeneSignal *(table 2). The second procedure uses the RMA algorithm [10,12] to convert the raw probe level data, stored in the *RGList *as *gMeanSignal *(table 2), into a summarized microRNA gene signal. Data pre-processing in the first protocol is accomplished by the following steps: 1) acquisition of the TGS processed by AFE; 2) normalization between arrays using the scale or quantile (default) methods. The AFE-TGS normally contains negative values for a few microRNAs, and these are converted before log transformation into positive signals, either by rounding up smaller values to 0.5 (default) or by adding the quantity |min (AFE-TGS)| + *offset*, where *ddTGS$R *is the matrix that contains the TGS and *offset *is an arbitrary positive constant chosen by the user. The RMA estimates a unique signal for each microRNA gene by fitting a linear model that takes into account the probe affinity effect. Robust estimates in the linear model are obtained by using the median polish algorithm. The RMA algorithm is applied in the following steps. 1) The raw mean signal is background corrected (optional) using the exponential + normal convolution model [18] in the *rma.background.correct *function of the Bioconductor *preprocessCore *package [20]. 2) The signal is normalized between arrays by quantile normalization; 3) The signals are log 2 transformed; 4) The median of the replicated probes (features with the same sequence) are obtained, normally resulting in 2, 3 or 4 different measures (probe level data) interrogating the same microRNA; 5) the probe level data are summarized into a single microRNA gene measure using the *rma_c_complete_copy *function of the *affy *package [21]. In both protocols, normalization between arrays is done with the *normalizeBetweenArrays *function from the *limma *package [16].

After obtaining the normalized total gene signal by using either of the two protocols, genes can be removed from the analysis by using the quality flags that AFE attaches to each feature (table 2). *AgiMicroRna *removes genes that are not expressed in any experimental condition. For a given feature *x*_*i *_across replicates, we set the minimum percentage (L) of features that must remain in at least one experimental condition with a flag indicating that the gene has been detected (AFE-flag = 1). Additionally, for a more stringent selection, all microRNAs can be removed whose expression level is close to that of the negative control features. As before, we set a limit for the percentage of microRNAs that must have a signal above a threshold expression value in at least one of the experimental conditions. This threshold expression value is defined internally in *AgiMicroRna *as the mean expression of the negative controls + 1.5 times the standard deviation of the negative control signals. Finally, the processed signal to be used for making statistical inferences is stored in an *ExpressionSet *object [14].

### Differential expression analysis

*AgiMicroRna *uses the linear model features implemented in the *limma *package [16] to fit a linear model to each microRNA, and thereby assesses the differential expression between different experimental conditions. The *AgiMicroRna *function *significantMicroRna *summarizes the results of the differential expression analysis and extracts information from the *MArrayLM *and *TestResults *objects generated by the *limma *functions. The function *significantMicroRna *creates a list of the genes with their related statistics (table 3). When multiple contrasts are made, the method used to select the significant genes in *limma *can be either *separated *or *nestedF *(see *decideTests *in the *limma *user guide for a detailed description on these two methods). When *separated *is plugged into the *significantMicroRna *function a list of all the genes that have been analyzed is generated for each contrast. These lists include the statistics obtained from the differential expression analysis. The html output files only include the links to the miRBase http://microrna.sanger.ac.uk/ for the microRNAs that have been declared as significant.

## Discussion

*AgiMicroRna *eases the progress of reading the scanned Agilent microRNA array data exported by AFE image analysis software into R for pre-processing (background correction, normalization, probe summarization, probe filtering, and quality control) and differential expression analysis. A key issue for potential users is likely to be the choice among the different data pre-processing alternatives included in the package. In this regard, Irizarry et al. (personal communication) compared the performance of different microRNA array platforms and found that background correction can increase the false positive detection of fold changes in low expressed microRNAs. Therefore the RMA method implemented in *AgiMicroRna *was designed to be used with or without background correction. In a related study [12], we showed that, for Agilent microRNA data, the use of the RMA algorithm without background correction reduced signal variability, especially for genes expressed at low intensity. However, the overall difference in variability between TGS and RMA was not large [12]. Therefore both the TGS normalized by quantiles and the RMA signal estimated without background correction are plausible options for the pre-processing of Agilent microRNA array data with the *AgiMicroRna *package.

## Conclusions

In this paper we present *AgiMicroRna*, a library of functions for the pre-processing and differential expression analysis of Agilent microRNA array data. *AgiMicroRna *allows the use of either the TGS processed by the Agilent image analysis software or the signal estimated by the RMA algorithm. Either TGS normalized by quantiles or the RMA algorithm used without background correction are reasonable alternatives for the pre-processing of Agilent microRNA data [12]. The program also includes a variety of graphics utilities to assess data quality. Differential expression is analyzed using functions from the Bioconductor *limma *package [16] and significant differential expression is assigned on the basis of the multiplicity of the tests. The package is integrated in the Bioconductor project [14] and uses standard objects to ensure compatibility with other packages. The software is provided with a manual containing full technical details and a set of guidelines to enable users to test the package functionality.

## Availability and requirements

The software is implemented as open-source and is accessible at the Bioconductor web site (http://www.bioconductor.org) under the GPL license.

## Competing interests

The authors declare that they have no competing interests.

## Supplementary Material

Additional file 1 **Supplementary material**. In this supplementary file we give the R code that can be used for the pre-processing and differential expression of your Agilent microRNA data files. Click here for file
