# Supplementary material for: Pre-processing and differential expression analysis of Agilent microRNA arrays using the AgiMicroRna Bioconductor library
Source: BMC Genomics. 2011 Jan 26;12:64. doi: 10.1186/1471-2164-12-64 (PMC3037903; doi:10.1186/1471-2164-12-64)
Supplement: Additional file 1 — Supplementary material. In this supplementary file we give the R code that can be used for the pre-processing and differential expression of your Agilent microRNA data files. [file 1471-2164-12-64-S1.DOC]

### In this supplementary file we give the R code that can be used for the pre-processing and differential expression analysis of your Agilent microRNA data files. First, we give the code for the pre-processing steps, and then for the differential expression analysis, using the functions included in the AgiMicroRna library. All these functions are explained in the *vignette file* of the package. This *vignette* document is a *pdf* file that contains a task-oriented description of the package functionality using examples that can be used interactively. Once you have installed and load the AgiMicroRna library in your R session, you can view the AgiMicroRna *vignette* entering at the R prompt: *browseVignettes(package = "AgiMicroRna")*

**Working with the AgiMicroRna library**

1) Open an R console and set your working directory path. Then load AgiMicroRna library by typing in an R console:

*setwd("C:/My_working_directory_path”)*

*library(AgiMicroRna)*

2) In the *PreProcessing.R* script (see below), set the parameters that you want to use in the pre-processing of your data and save the changes.

3) Launch the *PreProcessing.R* script (see below) by typing in an R console:

*source(“PreProcessing.R”)*

### 1.- R script for the pre-processing of Agilent MicroRNA data

Here we give an R code that includes a sequential call to the specific functions of the AgiMicroRNA library for the pre-processing of your data files. Save this R code in a text file, using, for example, the name *PreProcessing.R*

***# PreProcessing.R***

***# OVERALL parameters***

*# QC-PLOTS*

*makeQCplots.v1=FALSE*

*makeQCplots.v2 = FALSE*

*makePROBES.CV=FALSE*

*foreground="MeanSignal"*

***# PRE-PROCESSING***

*AFE.TGS = TRUE*

*RMA.TGS = FALSE*

***##### for AFE.TGS***

*half=TRUE # ddTGS signal with 'half method'*

*offset=5*

*makePLOT=FALSE*

***# NORMALIZATION of ddTGS***

*NORMmethod="quantile"*

*makePLOTpre=TRUE*

*makePLOTpost=TRUE*

***##### for RMA.TGS***

*normalize = TRUE*

*background = FALSE*

*makeRMA.plots = FALSE*

***# FILTERING PROBES***

*control = TRUE*

*IsGeneDetected = TRUE*

*wellaboveNEG = FALSE*

*limIsGeneDetected = 50*

*limNEG = 25*

*makePLOT = FALSE*

***# CREATING & WRITING EXPRESIONSET***

*makePLOT =FALSE*

***# READING THE Target File***

*targets=readTargets(infile="targets.txt",verbose=TRUE)*

***# READING THE DATA (RGList)***

*dd=readMicroRnaAFE(targets,verbose=TRUE)*

*names(dd)*

***# QC-PLOTS***

*if(makeQCplots.v1){*

*qcPlots(dd, offset = 5, MeanSignal = TRUE, ProcessedSignal = FALSE,*

*TotalProbeSignal = FALSE, TotalGeneSignal = FALSE, BGMedianSignal = FALSE,*

*BGUsed = FALSE, targets)*

*}*

*# The same plots can be also generated calling the corresponding functions*

*# individually. Next we show how to use these functions using the gMeanSignal. This signal has been stored one of the components of the uRNAList: dd$meanS*

*if(makeQCplots.v2){*

*boxplotMicroRna(log2(dd$meanS), maintitle = "log2 Mean Signal", colorfill = "orange")*

*plotDensityMicroRna(log2(dd$meanS), maintitle = "log2 Mean Signal")*

*op=par(mfrow=c(1,1),ask=TRUE)*

*ddaux = dd*

*ddaux$meanS = log2(dd$meanS)*

*mvaMicroRna(ddaux, maintitle = "log2 Mean Signal", verbose = FALSE)*

*rm(ddaux)*

*par(op)*

*RleMicroRna(log2(dd$meanS), maintitle = "log2 Mean Signal - RLE")*

*hierclusMicroRna(log2(dd$meanS), targets$GErep, methdis = "euclidean",*

*methclu = "complete", sel = TRUE, 100)*

*}*

***# REPLICATED PROBES: Coefficient of Variation***

*if(makePROBES.CV){*

*cvArray(dd, foreground = "MeanSignal", targets, verbose = TRUE)*

*}*

***# PRE-PROCESSING***

***# USING AFE gTotalGeneSignal & # NORMALIZATION***

*#* ***tgsMicroRna:*** *creates an uRNAList object that contains the Total Gene Signal computed*

*# by the Agilent Feature Extraction algorithms*

*#* ***tgsNormalization****: creates an uRNAList object containing the Normalized Total*

*# Gene Signal in log 2 scale*

*if(AFE.TGS){*

*message('pre-processing: AFE TGS')*

*cat('\n')*

*ddTGS = tgsMicroRna(dd, half = TRUE, makePLOT = FALSE,verbose = FALSE)*

*ddNORM = tgsNormalization(ddTGS, "quantile", makePLOTpre = FALSE,*

*makePLOTpost = FALSE, targets, verbose = TRUE)*

*}*

***# RMA***

*#* ***rmaMicroRna:*** *creates an uRNAList output that contains Total Gene Signal (TGS)*

*# computed by the RMA algorithm. This signal is in log2 scale*

*if(RMA.TGS){*

*message('pre-processing: RMA TGS')*

*cat(' normalize: ',normalize,'\n')*

*cat(' background: ',background,'\n')*

*cat('\n')*

*ddTGS.rma = rmaMicroRna(dd, normalize = TRUE, background = FALSE)*

*}*

*# To get some plots with the gene signal processed by RMA*

*if(makeRMA.plots){*

*MMM=ddTGS.rma$TGS*

*colnames(MMM)=colnames(dd$TGS)*

*maintitle='TGS.rma'*

*colorfill='blue'*

*ddaux=ddTGS.rma*

*ddaux$G=MMM*

*op=par(mfrow=c(1,1),ask=TRUE)*

*## MA plot - distinguishes between different features ##*

*mvaMicroRna(ddaux,maintitle,verbose=TRUE)*

*rm(ddaux)*

*## MA plot - does not distinguish between different features ##*

*mvaBASIC(MMM)*

*par(op)*

*RleMicroRna(MMM,"RLE TGS.rma",colorfill)*

*boxplotMicroRna(MMM,maintitle,colorfill)*

*plotDensityMicroRna(MMM,maintitle)*

*}*

***# FILTERING PROBES***

*if(AFE.TGS){*

*ddPROC = filterMicroRna(ddNORM,*

*dd,*

*control = TRUE,*

*IsGeneDetected = TRUE,*

*wellaboveNEG = FALSE,*

*limIsGeneDetected = 50,*

*limNEG = 25,*

*makePLOT = FALSE,*

*targets,*

*verbose = TRUE)*

*}*

*if(RMA.TGS){*

*ddPROC = filterMicroRna(ddTGS.rma,*

*dd,*

*control = TRUE,*

*IsGeneDetected = TRUE,*

*wellaboveNEG = FALSE,*

*limIsGeneDetected = 50,*

*limNEG = 25,*

*makePLOT = FALSE,*

*targets,*

*verbose = TRUE)*

*}*

***# CREATING EXPRESIONSET OBJECT***

*esetPROC = esetMicroRna(ddPROC, targets, makePLOT = FALSE, verbose = TRUE)*

***# WRITING EXPRESIONSET OBJECT: ProcessedData.txt***

*writeEset(esetPROC, ddPROC, targets, verbose = TRUE)*

### 2.- R script for the differential expression of Agilent MicroRNA data

Once you have the pre-processed data stored in an *esetPROC* object, you can proceed to the differential expression analysis:

1) Keep the *DifferentialExpression.R* script (below) in a file and save it using, for example, the name *DifferentialExpression.R*

2) Set the parameters for your differential expression analysis and save the changes.

3) Launch the script by typing in an R console:

*source(“DifferentialExpression.R”)*

Here is the *DifferentialExpression.R* script

***## DifferentialExpression.R Script***

***# parameters***

*PVcut=0.05*

*Mcut=0.0*

*MTestmethod = "BH"*

*DEmethod="separate"*

***# factors to be used in the linear model***

*levels.treatment=levels(factor(targets$Treatment))*

*treatment=factor(as.character(targets$Treatment),*

*levels=levels.treatment)*

***# design matrix***

*design=model.matrix(~ -1 + treatment )*

***# contrast matrix***

*CM=cbind(GrasavsHueso=c(1,-1,0),*

*NDFvsGrasa=c(-1,0,1),*

*NDFvsHueso=c(0,-1,1))*

***# linear model***

*fit2 = basicLimma(esetPROC, design, CM, verbose = TRUE)*

***# significant genes***

*DE = getDecideTests(fit2,*

*DEmethod = DEmethod,*

*MTestmethod = MTestmethod,*

*PVcut = PVcut,*

*verbose = TRUE)*

*pvalHistogram(fit2,*

*DE,*

*PVcut = PVcut,*

*DEmethod = DEmethod,*

*MTestmethod = MTestmethod,*

*CM,*

*verbose = TRUE)*

*significantMicroRna(esetPROC,*

*ddPROC,*

*targets,*

*fit2,*

*CM,*

*DE,*

*DEmethod = DEmethod,*

*MTestmethod = MTestmethod,*

*PVcut = PVcut,*

*Mcut = Mcut,*

*verbose = TRUE)*
